# Maternal and Infant Factors Associated with Human Milk Oligosaccharides Concentrations According to Secretor and Lewis Phenotypes

**DOI:** 10.3390/nu11061358

**Published:** 2019-06-17

**Authors:** Karina M. Tonon, Mauro B. de Morais, Ana Cristina F. V. Abrão, Antonio Miranda, Tania B. Morais

**Affiliations:** 1 Nutrition Postgraduate Program, Universidade Federal de São Paulo, São Paulo 040024-002, Brazil; karina.tonon@unifesp.br; 2 Division of Pediatric Gastroenterology, Universidade Federal de São Paulo, São Paulo 04020-040, Brazil; maurobmorais@gmail.com; 3 Breastfeeding Incentive and Support Center, Universidade Federal de São Paulo, São Paulo 04037-001, Brazil; ana.abrao@unifesp.br; 4 Department of Biophysics, Universidade Federal de São Paulo, São Paulo 04044-020, Brazil; miranda.unifesp@gmail.com; 5 Food Quality Control Laboratory, Universidade Federal de São Paulo, São Paulo 040024-002, Brazil

**Keywords:** human milk oligosaccharides, human milk composition, bioactive components, exclusive breastfeeding, Secretor phenotype, Lewis phenotype

## Abstract

Human milk oligosaccharides (HMOs) are multifunctional carbohydrates naturally present in human milk that act as prebiotics, prevent pathogen binding and infections, modulate the immune system and may support brain development in infants. HMOs composition is very individualized and differences in HMOs concentrations may affect the infant’s health. HMOs variability can be partially explained by the activity of Secretor (*Se*) and Lewis (*Le*) genes in the mother, but non-genetic maternal factors may also be involved. In this cross-sectional, observational study, 78 single human milk samples ranging from 17 to 76 days postpartum (median: 32 days, IQR: 25–46 days) were collected from breastfeeding Brazilian women, analyzed for 16 representative HMOs by liquid chromatography coupled to mass spectrometry and associations between maternal and infant factors with HMOs concentrations were investigated. HMOs concentrations presented a high variability even in women with the same *SeLe* phenotype and associations with maternal allergic disease, time postpartum and with infant’s weight, weight gain and sex. Overall, we present unprecedented data on HMOs concentrations from breastfeeding Brazilian women and novel associations of maternal allergic disease and infant’s sex with HMOs concentrations. Differences in HMOs composition attributed to maternal *SeLe* phenotype do not impact infant growth, but higher concentrations of specific HMOs may protect against excessive weight gain.

## 1. Introduction

Breastfeeding is the natural and best feeding type for infants, providing not only nutrition but improving the general health of the mother-infant dyad [1]. Besides the perfect balance of nutrients and water in an amount matching the infant’s needs, human milk contains a myriad of bioactive components, including immunoglobulins, hormones, oligosaccharides, and others [2]. Human milk oligosaccharides (HMOs) are a complex group of free glycans synthesized by the lactating mammary gland composing the third major solid fraction of human milk, after lactose and lipids [3,4]. Emerging evidence has shown that HMOs act as prebiotics [5,6,7], antimicrobials [8], prevent pathogen binding and infections [9,10,11], modulate the immune system [12] and also may support brain development [13,14].

HMOs are composed of the monosaccharides glucose (Glc), galactose (Gal), *N*-acetylglucosamine (GlcNAc), fucose (Fuc) and *N-*acetylneuraminic acid (Neu5Ac). So far, about 150 individual HMOs have been identified [15], yet approximately 90% of the HMOs fraction is composed of less than 20 different structures [16,17]. Nearly all HMOs contain lactose in the reducing end, which can be elongated by the addition of GlcNAc and Gal to form type 1 (Galβ1-3GlcNAc) or type 2 (Galβ1-4GlcNAc) chains in β1-3 or β1-6 linkages, producing core structures. Additionally, Fuc and Neu5Ac can be attached to the HMO core or directly to the lactose reducing end [18]. According to the monosaccharides present in the structure, HMOs can be classified into three main groups: 1. neutral core, containing Glc, Gal and GlcNAc; 2. neutral fucosylated, containing one or more Fuc units; and 3. acidic, containing one or more Neu5Ac units.

The composition and concentrations of HMOs are unique in the milk of each woman and strongly depend on the activity of the Secretor (*Se*) and Lewis (*Le*) genes in the mammary glands. *Se* and *Le* genes encode the enzymes α1-2-fucosyltransferase (FUT2) and α1-3/4-fucosyltransferase (FUT3), respectively, involved in the biosynthesis of fucosylated HMOs [19,20]. Mutations on the *Se* gene inactivate FUT2, and consequently, milk from non-secretor (Se−) women contain no or only traces of α1-2 fucosylated HMOs. Mutations on the *Le* gene inactivate FUT3, and consequently, milk from Lewis-negative (Le−) women contain no or only traces of α1-4 fucosylated HMOs [21]. Based on the activity of the FUT2 (*Se*) and FUT 3 (*Le*) enzymes in the lactating woman, HMOs composition can be classified into four phenotypes: 1. Se+Le+, the most common, containing α1-2 and α1-4 fucosylated HMOs, such as 2’-fucosyllactose (2’-FL) and lacto-N-difuco-hexaose I (LNDFH I); 2. Se−Le+, which contain α1-4 fucosylated HMOs, such as lacto-N-difuco-hexaose II (LNDFH II), but does not contain α1-2 fucosylated HMOs, such as 2’-FL, lacto-N-fucopentaose I (LNFP I), difucosyllacto-N-hexaose c (DFLNH c) and LNDFH I; 3. Se+Le−, which contain α1-2 fucosylated HMOs, such as 2’-FL and LNFP I, but does not contain α1-4 fucosylated HMOs, such as DFLNH c, LNDFH I and II and; 4. Se−Le−, the least common phenotype, containing neither α1-2 nor α1-4, but only α1-3 fucosylated HMOs, such as 3’-fucosyllactose (3’-FL) and difucosyl-para-lacto-N-neohexaose (DFpLNnH), which occur in all the four *SeLe* groups, since their synthesis apparently is not influenced by the *Se* and *Le* genes [22,23]. Besides the influence of the *Se* and *Le* genes in the composition of HMOs, great differences in HMOs concentrations occur in the milk of women with the same genetic background [24]. The variability within *SeLe* groups indicates that other factors besides the activity of *Se* and *Le* genes may be involved in HMOs biosynthesis influencing their concentrations in human milk. However, little is known about the influence of non-genetic factors on HMOs composition and concentrations and only a few studies have addressed this question. There is some conflicting evidence about the influence of gestational age and lactation time on HMOs concentrations [23,25,26]. Recently, maternal factors such as parity and body mass index (BMI), as well as environmental factors such as geographic location have been associated with HMOs concentrations [27,28,29].

The understanding of the influencing factors on HMOs composition and concentrations is important since some of the HMOs effects on infant health have been related to specific structures and usually in a dose-dependent manner. For example, infants whose mother’s milk had low concentrations of α1-2 fucosylated HMOs presented a higher incidence of Campylobacter, calicivirus and moderate-to-severe diarrhea than those whose mother’s milk contained higher concentrations of α1-2 fucosylated HMOs [11]. Higher concentrations of α1-2 fucosylated HMOs also have been related to a lower risk of allergy at 2 and 5 years of age in infants with high hereditary allergy risk [30]. A lower total HMOs concentration and a higher proportion of 3’-sialyllactose (3’-SL) have been correlated with higher HIV transmission in Zambian infants [31]. Higher total HMOs concentrations were also associated with reduced mortality in HIV-exposed uninfected Zambian infants [32]. Furthermore, disialyllacto-N-tetraose (DSLNT), an acidic HMO, prevents necrotizing enterocolitis (NEC) in rats and lower amounts of DSLNT in human milk may predict the risk of NEC in preterm infants [33,34]. Furthermore, infant weight, body composition and nutritional status also have been associated with concentrations of specific HMOs [35,36].

Bearing this in mind, we developed this cross-sectional, observational study to measure absolute HMOs concentrations in a cohort of Brazilian mothers, characterize HMOs profiles and identify maternal and infant factors associated with HMOs concentrations.

## 2. Materials and Methods

### 2.1. Study Design

A total of 78 mature human milk samples were analyzed in the study. Each mother provided a single human milk sample. Participants of the study were mothers and their infants who attended the Centro de Incentivo e Apoio ao Aleitamento Materno (Breastfeeding Incentive and Support Center) (CIAAM) of the Universidade Federal de São Paulo (UNIFESP) between December 2013 and October 2015. Healthy full-term (gestational age ≥ 37 weeks), singleton, exclusively breastfed infants with 17 to 76 days of life (median: 32 days, IQR: 25–46 days) were included in the study. Infants that received antibiotic treatment, probiotics, water or any other food besides human milk were not included.

Clinical and demographic data were obtained from the CIAAM medical records of the mother-infant pairs and through a face-to-face interview with the mother. Maternal weight and height and infant weight and length were measured at inclusion. Maternal overweight and obesity were defined as BMI (kg/m^2^) > 25.0 and > 30.0, respectively. The socioeconomic status of the families was obtained through a standardized questionnaire for the economic classification in Brazil developed by the Brazilian Association of Research Companies [37]. Considering that there is no validated instrument for the screening of allergic diseases in adults, the International Study of Asthma and Allergies in Childhood (ISAAC) questionnaire was used. This questionnaire was translated and validated in the Portuguese language for application in children and adolescents [38,39,40].

Human milk samples were obtained by manual expression of the breast opposite to the one previously emptied by the infant, as informed by the mother. A CIAAM nurse performed the milk expression when the mother could not do it by herself. With the use of gloves, after hand hygiene, massaging of the breast and the discard of the first drops, 5 to 15 mL of human milk were collected in a sterilized glass bottle and transferred to a Falcon polypropylene tube. All human milk samples were collected in the morning (8:30–12:00 a.m.) and stored at −20 °C until HMOs analysis. Data and human milk sample collection were carried out on the same day at CIAAM. 

### 2.2. Ethical Considerations

This study was approved by the Ethics Committee of UNIFESP (protocol No. 419.162) and complied with the Declaration of Helsinki. All lactating women received detailed oral and written information about the study and voluntarily agreed to participate. Written informed consent was obtained from each participant prior to the data and sample collection.

### 2.3. HMOs Analysis

HMOs were extracted from human milk, identified and quantified by liquid chromatography-mass spectrometry (LC-MS) following a validated method previously reported in detail [41]. Analyses were performed in duplicate for each human milk sample and HMOs concentrations were reported as the mean value of the duplicates. Absolute quantification was carried out for 16 representative HMOs, including 4 neutral core (lacto-N-tetraose (LNT); lacto-N-neotetraose (LNnT); lacto-N-hexaose (LNH); lacto-N-neohexaose (LNnH)), 7 neutral fucosylated (2’-fucosyllactose (2’-FL); 3’-fucosyllactose (3’-FL); lacto-N-fucopentaose I (LNFP I); lacto-N-difuco-hexaose I (LNDFH I); lacto-N-difuco-hexaose II (LNDFH II); difucosyl-para-lacto-N-neohexaose (DFpLNnH); difucosyllacto-N-hexaose (c) (DFLNH c)) and 5 acidic HMOs (3’-sialyllactose (3’-SL); 6’-sialyllactose (6’-SL); LS-tetrasaccharide a (LSTa); LS-tetrasaccharide b (LSTb); LS-tetrasaccharide c (LSTc)). Analytical standards of the 16 HMOs were purchased from Dextra Laboratories (Reading, UK). The 16 HMOs included in the analysis are within the most abundant HMOs [42] and are expected to represent about 90% of the total HMOs in human milk [27]. The criteria utilized to select the HMOs to be analyzed were: (a) availability of quantitative data in the literature; (b) structures reported among the most abundant in human milk; (c) structures that allowed the determination of the *SeLe* phenotype and (d) commercial availability of analytical standards.

#### 2.3.1. HMOs Extraction

Aliquots of 100 µL of the human milk samples were defatted via centrifugation at 5000× *g* for 15 min at room temperature; then 50 µL was diluted with 950 µL Milli-Q water and homogenized in vortex. Proteins were removed by ultrafiltration, transferring 500 µL of the defatted diluted sample to an Amicon 10 kDa (Merck, Darmstadt, Germany) molecular weight cutoff filter and centrifuging at 12,000 *g* for 30 min. The filtrate, which contained the HMOs extract, was collected and 100 µL was subjected to a reduction reaction with 100 µL of 0.25 M sodium borohydride (Sigma-Aldrich, Saint Louis, USA) at room temperature. The reaction was terminated after 30 min by the addition of 100 µL of 0.25 M acetic acid (JT Baker, Center Valley, USA). There was 20 µL of reduced extracts injected into the LC-MS system for identification and quantification of the 16 HMOs.

#### 2.3.2. HMOs Identification and Quantification by LC-MS

HMOs separation and quantification were performed on a Waters Alliance 2695 HPLC (Waters, Milford, USA) system equipped with a column heater set to 30 °C and a Hypercarb column (100 × 2.1 mm, 3 µm, Thermo Scientific, Waltham, USA) coupled to a Waters Micromass Quattro LC mass spectrometer equipped with an electrospray ionization (ESI) source. The software MassLynx 4.1 (Waters, Milford, USA) was used to control system components and for data acquisition and processing.

The mobile phases consisted of Milli-Q ultrapure water (A) and acetonitrile (B), both containing formic acid at 0.1% and delivered at a flow rate of 200 µL/min. HMOs separation was carried out over 55 min with a gradient consisting of an initial increase from 0 to 12% B over 21 min, followed by a second increase from 12 to 40% B over 11 min and a third increase from 40 to 100% B over 5 min. A washing step was conducted at 100% B for 5 min. The gradient was then decreased to 0% B over 1 min and maintained at 0% B for 12 min for column equilibration.

The ionization parameters were optimized with individual standard solutions of the 16 HMOs at 100 µg/mL in water injected directly into the MS. The optimized ionization conditions were as follows: capillary voltage at 3.0 kV, cone voltage at 30 V, cone gas flow at 86 L/h and the source, and desolvation temperatures at 150 °C and 250 °C, respectively. The dwell time was 0.055 min. The MS was operated with selected ion monitoring (SIM) in negative mode ([M-H]^−^) for all HMOs. The deprotonated ions [M-H]^−^ used for monitoring of neutral core HMOs were *m*/*z* 708.63 for LNT and LNnT and *m*/*z* 1073.96 for LNH and LNnH. The deprotonated ions [M-H]^−^ used for fucosylated HMOs monitoring were *m*/*z* 489.44 for 2’-FL and 3’-FL, *m*/*z* 854.77 for LNFP I, *m*/*z* 1000.91 for LNDFH I and LNDFH II and *m*/*z* 1366.25 for DFpLNnH and DFLNHc. The deprotonated ions [M-H]^−^ used for the monitoring of acidic HMOs were *m*/*z* 634.55 for 3’-SL and 6’-SL and *m*/*z* 999.88 for LSTa, LSTb, and LSTc.

HMOs absolute quantification was performed using a linear calibration curve (0.039–5 µg/mL) constructed from a standard solution containing all the 16 HMOs analytical standards at 5 µg/mL, in water, which was serially diluted to 0.039 µg/mL. The calibration curve presented excellent linearity (R^2^ ≥ 0.99) for all the 16 HMOs. The limit of quantification (LOQ) was established as the lower concentration capable of generating a signal to noise ratio (s/n) ≥ 10. LOQ was 0.039 µg/mL for all HMOs except LNFP I, for which it was 0.156 µg/mL.

### 2.4. Secretor and Lewis Phenotype Determination

The Secretor and Lewis phenotype of the mothers was determined based on the presence of indicative α1-2 and α1-4 fucosylated HMOs in the human milk sample, as previously done in other studies [22,23,25,26,43,44]. Women whose human milk sample presented both α1-2 and α1-4 fucosylated structures were classified as Secretor positive/Lewis positive (Se+Le+) and attributed to Group 1. Women who presented α1-4 but not α1-2 fucosylated HMOs were classified as Secretor negative/Lewis positive (Se−Le+) and assigned to Group 2. Those who presented α1-2 but not α1-4 fucosylated HMOs in their sample were classified as Secretor positive/Lewis negative (Se+Le−) and attributed to Group 3. When the human milk sample did not present neither α1-2 nor α1-4 fucosylated HMOs, the woman was classified as Secretor negative/Lewis negative (Se−Le−) and assigned to Group 4. For the classification according to the Secretor phenotype, women from the Groups 1 and 3 were grouped as secretor (Se+) and women from Groups 2 and 4 were assigned to the non-secretor (Se−) group. Table 1 shows the fucosylated HMOs profile in the human milk utilized to assign the Secretor and Lewis phenotype of the lactating mothers participating in the study.

### 2.5. Statistics

Descriptive statistics and statistical tests were performed with the use of the softwares Statistica 64, version 12 and R, version 3.4.4. As the normality assumption was not satisfied, a non-parametric Analysis of Covariance (Ranked ANCOVA or Quade test [45]) was employed to verify differences among the *SeLe* groups with postpartum days as a covariate and values of each of the HMOs as dependent variables. A free web-program [46] was used to perform the ranked ANCOVA (post-hoc Tukey-Kramer test). Analysis of Variance (ANOVA) and Kruskal-Wallis test were performed to verify associations between *SeLe* groups and quantitative clinical variables. Mann-Whitney, Student’s *t* test and Pearson’s chi-square were used to verify differences in HMOs concentrations, clinical and demographic characteristics between secretors and non-secretors. Fisher’s exact test was conducted to verify associations between *SeLe* groups, secretor status, and allergic diseases. Spearman rank correlation was used to verify correlations between HMOs concentrations and quantitative clinical variables. The association of clinical and demographic characteristics with HMOs concentrations was analyzed separately for each *SeLe* and *Se* phenotypes. Since in our cohort we had only one participant assigned to Group 4 (Se−Le−), she was not included in the statistical analyses. Therefore, statistical tests performed to verify differences among the *SeLe* groups were conducted considering only Group 1, Group 2 and Group 3. For the statistical analyses considering only the *Se* phenotype, the woman from Group 4 was included, assigned to the non-secretor group. All statistical analyses were considered significant at *p* < 0.05.

## 3. Results

### 3.1. Study Population

Table 2 shows the *SeLe* phenotypes distribution and characteristics of the mothers participating in the study. There were no significant statistical differences among *SeLe* groups for the studied variables. However, when considering *Se* phenotype alone, the incidence of allergic disease was significantly higher (Fisher’s exact test, *p* = 0.028) in women presenting Se+ phenotype (23/68) than in Se− women (0/10).

Table 3 presents the clinical characteristics of the infants according to their mothers’ *SeLe* phenotypes. From the total 78 infants, 37 (49%) were boys. Boys were 27 (47%) in Group 1; 4 (44%) in Group 2 and 5 (56%) in Group 3. The only one infant from Group 4 was a 37 days old boy. The range of the infants’ age was 17 to 76 (IQR: 27–48) days in Group 1; 20 to 50 (IQR: 24–41) in Group 2 and 20 to 62 (IQR: 22–32) in Group 3. Infant’s age corresponds to the day of milk sampling and presented a coefficient of variation (CV) of 37%, 35% and 42% in Groups 1, 2 and 3, respectively. There were no significant statistical differences among infants from *SeLe* groups for all the studied variables.

### 3.2. HMOs Composition and Concentrations

Table 4 shows the concentrations, expressed as mean, standard deviation (SD) and coefficient of variation of the HMOs concentrations according to the *SeLe* phenotypes and to the classification in fucosylated, neutral core, and acidic. HMOs concentrations were expressed as mean ± SD to facilitate comparisons with other studies, however, for statistical analysis non-parametric tests were used. Complete descriptive statistics as well the results of the sole sample of the Group 4 (Se−Le−) are available as Supplementary Materials.

HMOs concentrations demonstrated a great variability in the milk of women with the same *SeLe* phenotype, as expressed by the coefficient of variation (CV), which was higher than 20% for all the studied HMOs exceeding 100% in some cases. Fucosylated and neutral core HMOs presented the highest variation. Regarding individual HMOs, seven structures presented concentrations significantly different among the *SeLe* groups: 2’-FL, 3’-FL, LNFP I, LNDFH II, LNH, LNnH and LSTc.

Group 2 presented a significantly lower concentration of total and fucosylated HMOs when compared to Groups 1 and 3. Group 1 and Group 3 did not show differences in their concentrations either for total, fucosylated, acidic and neutral core HMOs. No differences were seen regarding total neutral core and total acidic HMOs among *SeLe* groups.

**Figure 1:**
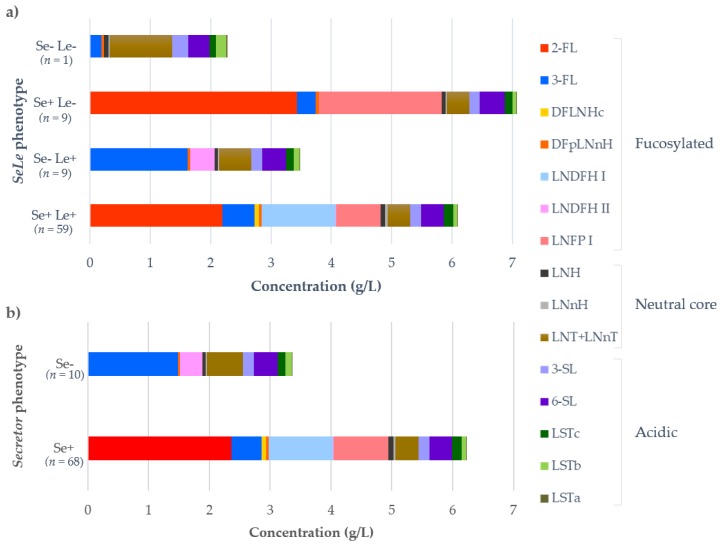
Human milk oligosaccharides (HMOs) profile and concentrations in human milk according to the *SeLe* (**a**) and *Se* (**b**) phenotype of the lactating mother.

Figure 1 illustrates the HMOs profile in the milk according to the *SeLe* and *Se* phenotype of the mother. *Se* phenotype was responsible for the major differences in the HMOs profile and concentrations, as can be noted in Figure 1a that Se*+* groups (Se+Le+ and Se+Le−) presented a clear different pattern—both in HMOs diversity and total concentration—from the Se− groups (Se−Le+ and Se−Le−). Main differences occur in the fucosylated HMOs profile. Se+ women had a significantly higher total fucosylated HMOs and total HMOs in the milk than Se− women (Mann-Whitney test, *p* < 0.0001). Neutral core and acidic HMOs profiles are quite similar among the *SeLe* and *Se* groups. Furthermore, there were no differences in total neutral core (Mann-Whitney test, *p* = 0.095) and total acidic HMOs (Mann-Whitney test, *p* = 0.565) between Se+ and Se− milk.

### 3.3. Associations of Maternal and Infant Factors with HMOs Concentrations in SeLe Groups

Women presenting allergic disease had significantly higher DFpLNnH concentrations than women without allergic disease both in Se+Le+ (0.05 and 0.04 g/L, respectively; Mann-Whitney test, *p* = 0.023) and Se+Le− (0.03 and 0.01 g/L, respectively; Mann-Whitney test, *p* = 0.028).

In Se+Le− milk, concentrations of some HMOs differed regarding the infant’s sex. Mothers of girls had a significantly higher 2’-FL concentration in their milk than mothers of boys (4.84 and 2.31 g/L, respectively; Mann-Whitney test, *p* = 0.016). However, mothers of boys had higher concentrations of LNH in their milk than mothers of girls (0.10 and 0.02 g/L, respectively; Mann-Whitney test, *p* = 0.016), as well as LNT+LNnT (0.54 and 0.17 g/L, respectively; Mann-Whitney test, *p* = 0.038) and total neutral core HMOs (0.67 and 0.20 g/L, respectively; Mann-Whitney test, *p* = 0.019). Maternal nutritional status, type of delivery, socioeconomic status and having pets at home had no associations with HMOs concentrations in any of the *SeLe* groups.

Table 5 and Table A1 and Table A2 (Appendix A) show the Spearman rank correlation coefficient (r) between HMOs concentrations and quantitative clinical variables by *SeLe* group. Several significant correlations (*p* < 0.05) occurred in all *SeLe* groups, especially negative correlations between acidic HMOs and postpartum days. 6’-SL and postpartum days presented a negative correlation in all three groups (Se+Le+, r = −0.71; Se−Le+, r = −0.70 and Se+Le−, r = −0.83). Postpartum days also had negative correlations with other acidic HMOs in Se+Le+ and Se−Le+ and with total acidic HMOs in Se+Le+ and Se+Le− (r = −0.79 and r = −0.74, respectively). Weak but significant positive correlations occurred between postpartum days and 3’-FL (r = 0.35) and LNDFH II (r = 0.27) in Se+Le+ (Table 5).

Negative correlations occurred among several HMOs and clinical variables of the infant, mainly in Se+Le+, where infant’s weight presented negative correlations with all the acidic HMOs, total acidic (r = −0.61), total neutral core (r = −0.33), total fucosylated (r = −0.27) and total HMOs (r = −0.39). Infants’ length and weight gain also had negative correlations with several HMOs, including total neutral core (infants’ length, r = −0.32), total fucosylated (weight gain, r = −0.32), total acidic (length, r = −0.35 and weight gain, r = −0.42) and total HMOs (weight gain, r = −0.37) in Se+Le+ (Table 5).

#### Associations Regarding *Se* Status Alone

Additionally, associations between clinical and demographic factors with HMOs concentrations were investigated considering *Se* phenotype alone, irrespective of the *Le* phenotype. Nutritional status was associated with concentrations of some HMOs in Se+ but not in Se− women. Se+ mothers with overweight (BMI 25–29.9 kg/m^2^) had a significantly higher concentration of 2’-FL (Dunn test, *p* = 0.030) and lower 3’-FL concentration (Dunn test, *p* = 0.011) than Se+ mothers with adequate weight (BMI 18.5–24.9 kg/m^2^). Se+ mothers with obesity (BMI > 30.0 kg/m^2^) presented no difference in 2’-FL and 3’-FL concentrations from Se+ mothers with adequate weight or overweight (Dunn test, *p* > 0.05). Allergic disease of the mother, infant’s sex, socioeconomic status and having pets at home were not associated with HMOs concentrations neither in Se+ nor in Se− women.

Table A3 and Table A4 (Appendix A) show the Spearman rank correlation coefficient (r) between HMOs concentrations and quantitative clinical variables by *Se* phenotype. Major significant correlations (*p* < 0.05) were observed in Se+ women, which presented mostly negative correlations of several HMOs with postpartum days and infants’ clinical variables. In Se+ women, some positive correlations were also observed, for instance, between 2’-FL and maternal BMI (r = 0.30) and between 3’-FL and postpartum days (r = 0.31). In the Se− group, significant correlations were mostly negative, involving both 6’-SL and LSTc with postpartum days and infants’ clinical variables.

## 4. Discussion

There are currently a few studies reporting HMOs absolute concentrations obtained by state-of-the-art analytical methods—such as LC-MS—in well-defined human milk samples [47]. Knowing the exact amount of the most representative HMOs is important to access the daily intake of HMOs by the infants and the biological effects of these molecules, since some HMOs may act in a dose-dependent manner [31,32,34,48,49]. In this study, we presented absolute concentrations of 16 representative HMOs measured by LC-MS from 78 full-term, mature human milk samples classified according to the *SeLe* phenotype and investigated associations between maternal and infant characteristics and HMOs composition/concentrations.

The frequency of the *SeLe* phenotypes varies among different ethnic populations. In Caucasians and Asians, the Se+Le+ phenotype (determined from human milk and blood samples) varies from 55 to 73%, Se−Le+ varies from 20 to 31%, Se+Le− varies from 6 to 11% and Se−Le− varies from 3 to 5% [25,27,50,51]. In African population from Burkina Faso, the *SeLe* distribution (from saliva samples) was reported to be 54% (Se+Le+), 14% (Se−Le+), 25% (Se+Le−) and 7% (Se−Le−) [52]. A study in a semi-isolated Black community in Northern Brazil reported a very similar *SeLe* phenotypes (from saliva and blood samples) distribution to Burkina Faso [53]. A study conducted in the state of São Paulo, Brazil [54] with 827 participants regardless of ethnicity revealed a prevalence of Se+Le+ phenotype (from blood samples) in 78% of the population, similarly with the Se+Le+ prevalence in our cohort (76%; from human milk samples), which was also composed by different ethnicities. Our cohort presented a *SeLe* distribution closer to the Caucasian/Asian than the semi-isolated Black from Northern Brazil. In our cohort, only one lactating mother was assigned to Group 4 (Se−Le−), which agrees with the rare occurrence of this phenotype in Caucasians and Asians [22,27].

Our study demonstrated a difference in HMOs profile and concentrations among *SeLe* groups, supporting previously reported results [23,25,26,27,55]. Furthermore, our results showed that the different HMOs composition of the 3 *SeLe* groups had no influence on infant’s weight gain and anthropometric parameters, which reinforces that human milk is nutritionally adequate, apart from the different HMOs composition. Similarly, Sprenger et al. (2017) did not observe differences in anthropometric parameters between infants who consumed breast milk with low or high 2’-FL content (Se− and Se+ milk, respectively) [56]. However, as in the study of Sprenger et al. (2017), our cohort was composed of healthy term-born infants. Charbonneau et al. (2016) reported a difference in HMOs composition from Se− mothers having healthy or severely stunted infants, which was not observed in Se+ mothers [36]. Therefore, the influence of *SeLe* milk groups on other populations, such as preterm or malnourished infants remains to be studied.

In a systematic review of 21 studies on HMOs concentrations, Thurl et al. (2017) reported TF-LNH, 2’-FL, DF-LNH II, and LNFP I to be the most abundant neutral HMOs and 6’-SL the most abundant acidic HMO in Se+ term human milk. However, the authors warned about a possible bias on the high concentrations of TF-LNH and DF-LNH II, which needs to be confirmed [47]. The systematic review reported concentrations of 33 HMOs and the results corroborate that the 16 HMOs analyzed in our study are among the most representative in human milk, except for DFLNHc and DFpLNnH (in term milk), which were not reported in the systematic review [47]. Our results largely agree with the concentrations reported in the above-mentioned systematic review and with Kunz et al. (2017), not included in the review [26]. The most abundant HMO in Se+Le+ e Se+Le− was 2’-FL, followed by LNDFH I in Se+Le+ and by LNFP I in Se+Le−. Among the acidic HMOs, we also observed the highest concentration of 6’-SL not only in Se+ but also in all *SeLe* groups. However, Kunz et al. (2017) reported a higher LNT concentration in Se−Le+ than in Se+Le+ and Se+Le−, which was not observed in our cohort.

Although our samples were collected in a single time point, we could observe some of the dynamics in HMOs concentrations during lactation reported in longitudinal studies, investigating correlations of HMOs concentrations and time postpartum. A significant increase in 3’-FL concentrations during the first months of lactation, as well as a significant decrease in LNFP I and LSTc have been previously reported [23,29,47,57,58,59,60]. We observed a significant positive correlation between 3’-FL and time postpartum and significant negative correlations between several HMOs—including LNFP I and LSTc—and time postpartum in Se+Le+ milk (Table 5). As in the study of McGuire et al. (2017) [28], 6’-SL, LSTc and LNH negative correlations with time postpartum were also observed. Total acidic HMOs presented the highest negative Spearman rank correlation coefficient (r = −0.79) with time postpartum in Se+Le+, which is in accordance with a significant decrease of acidic HMOs in the first three months of lactation reported in the systematic review of HMOs concentrations [47]. However, although associated with the variability in the concentrations of some HMOs, time postpartum does not explain variability alone. For example, the HMO with the highest coefficient of variation in Se+Le+ milk was LNDFH II (CV 293%, Table 4), but the correlation of LNDFH II concentrations with time postpartum had a low correlation coefficient (r = 0.27, Table 5), although significant. This is even more evident with DFpLNnH in Se+Le+, with a CV of 154% (Table 4), but without significant correlation with time postpartum (r = 0.01, Table 5).

There are some hypotheses about the role of HMOs on infant allergy development, related to the establishment of the gut microbiota, but the influence of maternal allergic diseases on HMOs composition and concentrations have not been substantially studied [30,61,62,63]. In our study, we investigated whether maternal allergic disease (asthma, rhinitis or eczema) could explain, at least in part, the great variability in the concentrations of HMOs observed in each *SeLe* milk group. We observed significantly higher DFpLNnH concentrations in the milk from Se+Le+ and Se+Le− women with allergic disease than in those without allergic disease. When we considered secretor status alone, there were no differences in DFpLNnH concentrations or another HMO between the groups. Sjögren et al. (2007) [62] performed—in a smaller cohort—the only study previously published that investigated associations between maternal allergic diseases and HMOs concentrations and found no differences in the concentrations of nine neutral HMOs from colostrum of allergic and non-allergic mothers. However, DFpLNnH was not included in the analysis [62]. Furthermore, in our cohort, Se+ mothers presented a higher prevalence of allergic disease than Se− mothers, which agrees with a higher prevalence of asthma and a higher susceptibility to asthma exacerbation observed in Se+, particularly in blood group O/Se+ individuals [64,65]. On the other hand, a 1968 study reported no differences in the incidence of allergic diseases between Se+ and Se− individuals [66]. Similarly, Sprenger et al. (2017) did not observe differences in allergy prevalence on mothers presenting or not 2’-FL in the milk (Se+ and Se−, respectively) [30].

We observed differences in 2’-FL and 3’-FL concentrations between overweight and eutrophic Se+ mothers, as well as a positive correlation between 2’-FL concentrations in Se+ milk and maternal BMI. However, the difference in 2’-FL and 3’-FL concentrations from mothers with distinct nutritional statuses was no longer significant when considering the Lewis phenotype together. Positive correlations between maternal BMI or weight and total HMOs or 2’-FL concentrations have been previously reported [27,28]. It has been hypothesized that maternal diet may influence HMOs concentrations, yet Azad et al. (2018) found no associations between the overall diet quality and HMOs concentrations [29]. More studies are needed to verify dietary and nutritional effects on HMOs composition and concentrations.

To date, few studies have investigated associations between parity and HMOs concentrations, without agreement. While Elwakiel et al. (2018) found no associations [27], Azad et al. (2018) observed higher LNT and LNnT and lower 3’-FL concentrations in multiparous mothers [29]. Interestingly, we observed a significant positive correlation between parity and some HMOs—including LNT+LNnT—and a negative correlation between parity and 3’-FL, similarly to Azad et al. (2018), but only in Se+Le− mothers (Table A2). However, Se+Le− was a small group, which may limit conclusions. We found no correlations between HMOs concentrations and parity in other *SeLe* groups.

We observed unprecedented significant differences (*p* < 0.04) in some HMOs concentrations between Se+Le− milk from mothers of boys and girls, even with a small number of mother-infant pairs in Se+Le− group. As previously reported, we found no associations between HMOs concentrations and socioeconomic status as well as with the type of delivery in any *SeLe* group [27,29,59].

Exclusively breastfed infants present lower weight gain—although adequate—during the first year of life than formula-fed infants [67,68]. There is a possible effect of breastfeeding on obesity prevention, related to the lower weight gain in the first year of life, but the mechanisms involved in this protection are not yet understood [69]. Importantly, the intricate composition and high amounts of HMOs are responsible for crucial differences between human milk and infant formula, which may contribute to the differences in health outcomes between breastfed and formula-fed infants [4,70]. HMOs may influence infant growth and the development of obesity directly since they are absorbed into the circulation and can induce systemic effects [71,72]. A study demonstrated that the HMO lacto-N-fucopentaose III modulates metabolic functions by improving glucose tolerance, insulin sensitivity and suppressing lipogenesis in the liver of diet-induced obese mice [73]. Additionally, HMOs may also modulate infant growth and obesity development indirectly by altering the gut microbiome [36,74]. Inverse associations between concentrations of individual HMOs and infant body composition have been previously reported. Alderete et al. (2015) observed that each 1 µg/mL increase in LNFP I concentration was associated with a 0.4 g lower infant weight at 1 month postpartum. At 6 months, inverse associations were also observed between LNFP I and body weight, lean and fat mass, as well as between LNnT and fat mass [35]. A higher proportion of LSTc in milk was associated with a lower infant’s weight-for-age (WAZ) score in the study conducted by Davis et al. (2016), who also observed that a higher proportion of 3’-SL, LNFP I + III and DFLNHa contributed positively to WAZ and height-for-age scores [75]. In our study, although the infants—all exclusively breastfed—presented adequate weight gain, we demonstrated inverse associations between concentrations of specific HMOs in breast milk—including LNFP I, LNT+LNnT and LSTc —and infant weight and weight gain at 1 month after birth (Table 5). The observed inverse associations between HMOs concentrations and infant weight gain suggest a potential role of HMOs on infant growth and metabolism, which deserves future investigations.

Among the major strengths of our study are the accurate, extensively validated method utilized for HMOs quantification and the standardized human milk collection procedure, which occurred in short time intervals to minimize natural diurnal variations and biases caused by milk sampling. Another important strength of our study is that our population was composed by exclusively breastfed infants, since the consumption of solid foods and water besides breastfeeding can impact infant growth, adding some bias on the results about the influence of human milk components on infant’s health. However, our study has some limitations. Although we selected 16 representative HMOs from the ~150 different structures occurring in human milk, it is possible that maternal and infant factors are associated with other HMOs not included in our analyses. The utilization of the ISAAC questionnaire for the screening of maternal allergy is also considered a limitation, however, there is no validated instrument available to be applied in the adult population. Furthermore, the small number of Se−Le+, Se+Le−, and Se−Le− mothers impaired more robust conclusions regarding these groups.

## 5. Conclusions

Our study reveals unprecedented data on HMOs composition from breastfeeding Brazilian women, as well as novel associations of maternal allergic disease and infant’s sex with concentrations of specific HMOs. Our results also showed that maternal and infant factors might influence HMOs concentrations in some *SeLe* group but not in others. For instance, the infant’s sex was associated with specific HMOs only in Se+Le−. Ultimately, the results showed that different HMOs composition does not impact infant growth, but higher concentrations of specific HMOs may protect against excessive weight gain.

## Figures and Tables

**Table 1:** Fucosylated Human milk oligosaccharides (HMOs) profile utilized to assign the Secretor and Lewis phenotype of the lactating mothers.

HMO	Structure	Fucose Linkages	Group 1: Se+Le+	Group 2: Se−Le+	Group 3: Se+Le−	Group 4: Se−Le−
2’-FL	Fucα1-2Galβ1-4Glc	α1-2	+	-	+	-
LNFP I	Fucα1-2Galβ1-3GlcNAcβ1-3Galβ1-4Glc	α1-2	+	-	+	-
LNDFH I	Fucα1-2Galβ1-3(Fucα1-4)GlcNAcβ1-3Galβ1-4Glc	α1-2 α1-4	+	-	-	-
DFLNH c	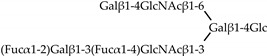	α1-2 α1-4	+	-	-	-
LNDFH II	Galβ1-3(Fucα1-4)GlcNAcβ1-3Galβ1-4(Fucα1-3)Glc	α1-3 α1-4	+	+	-	-
DFpLNnH	Galβ1-4(Fucα1-3)GlcNAcβ1-3Galβ1-4(Fucα1-3)GlcNAcβ1-3Galβ1-4Glc	α1-3	+	+	+	+
3’-FL	Galβ1-4(Fucα1-3)Glc	α1-3	+	+	+	+

Fuc: fucose; Gal: galactose; Glc: glucose; GlcNAc: *N*-acetylglucosamine.

**Table 2:** Descriptive characteristics of the mothers according to Secretor and Lewis phenotypes.

Variables	Group 1	Group 2	Group 3
	Se+ Le+	Se− Le+	Se+ Le−
	*n* = 59 (75.6%)	*n* = 9 (11.5%)	*n* = 9 (11.5%)
	Mean ± SD	Mean ± SD	Mean ± SD
Age, years	31 ± 7	29 ± 7	27 ± 4
BMI, kg/m^2^			
At inclusion	27 ± 5	26 ± 4	28 ± 5
Pre-gestational	25 ± 5	24 ± 4	26 ± 6
Parity, *n*	2 ± 1	2 ± 1	1 ± 0
Postpartum, days	38 ± 14	32 ± 11	31 ± 13
	*n* (%)	*n* (%)	*n* (%)
Cesarean delivery	32 (54)	6 (67)	2 (22)
Allergic disease, yes ^a^	19 (32)	0 (0)	4 (50)
Pets, yes ^b^	18 (38)	3 (38)	3 (43)
Socioeconomic status			
Class A (*n* = 6)	4 (7)	1 (11)	1 (11)
Class B (*n* = 45)	34 (58)	5 (56)	6 (67)
Class C (*n* = 26)	21 (36)	3 (33)	2 (22)
Education ^c^			
Elementary school (*n* = 6)	6 (12)	0 (0)	0 (0)
High school (*n* = 31)	22 (42)	5 (63)	4 (50)
Graduate (*n* = 24)	17 (33)	3 (38)	4 (50)
Postgraduate (*n* = 7)	7 (13)	0 (0)	0 (0)

^a^ Missing data from one mother of the group 3; ^b^ Missing data from 11 mothers of group 1; 1 mother from group 2 and 2 mothers from group 3; ^c^ Missing data from 7 mothers from group 1; 1 mother from group 2 and 1 mother from group 3.

**Table 3:** Descriptive characteristics of the infants according to the Secretor and Lewis phenotypes of their mothers.

Variables	Group 1	Group 2	Group 3
	Se+ Le+	Se− Le+	Se+ Le−
	*n* = 59 (75.6%)	*n* = 9 (11.5%)	*n* = 9 (11.5%)
	Mean ± SD	Mean ± SD	Mean ± SD
Age, days	38 ± 14	32 ± 11	31 ± 13
Gestational age, weeks	39 ± 1	39 ± 1	39 ± 1
Weight at birth, g	3237 ± 392	3358 ± 579	3173 ± 434
Weight at inclusion, g	4262 ± 764	4448 ± 1052	4148 ± 730
Length at inclusion, cm	54 ± 3	54 ± 3	51 ± 6
Weight gain, g/day ^a^	26 ± 16	24 ± 16	25 ± 6

^a^ Weight gain = (weight at inclusion − weight at birth)/age; *p* > 0.05 for all variables.

**Table 4:** HMOs concentrations in term mature human milk samples (*n* = 77) according to the *SeLe* phenotypes of the lactating women.

HMOs	Concentration (g/L)			Statistical Analysis ^a^	Multiple Comparison Analysis ^b^
	Group 1	Group 2	Group 3		
	Se+ Le+	Se− Le+	Se+ Le−		
	Mean ± SD	Mean ± SD	Mean ± SD		
	(CV, %)	(CV, %)	(CV, %)		
*Fucosylated*					
2’-FL	2.20 ± 0.98 (45)	–	3.43 ± 1.75 (51)	*p* = 0.019	
3’-FL	0.53 ± 0.33 (63)	1.62 ± 0.42 (26)	0.31 ± 0.58 (189)	*p* < 0.001	G3 < G1 < G2
LNFP I	0.73 ± 0.52 (71)	–	2.03 ± 1.51 (75)	*p* = 0.015	
LNDFH I	1.22 ± 0.81 (66)	–	–	–	
LNDFH II	0.02 ± 0.05 (293)	0.41 ± 0.22 (55)	–	*p* < 0.001	
DFLNHc	0.08 ± 0.05 (71)	–	–	–	
DFpLNnH	0.04 ± 0.07 (154)	0.04 ± 0.03 (72)	0.05 ± 0.10 (184)	*p* = 0.702	
*Neutral core*					
LNH	0.08 ± 0.06 (75)	0.05 ± 0.06 (116)	0.07 ± 0.06 (94)	*p* = 0.013	G1 = G3; G2 = G3; G2 < G1
LNnH	0.04 ± 0.03 (85)	0.02 ± 0.03 (151)	0.02 ± 0.02 (87)	p = 0.010	G2 = G3; G1 = G3; G2 < G1
LNT + LNnT	0.38 ± 0.17 (46)	0.54 ± 0.25 (47)	0.37 ± 0.25 (67)	*p* = 0.146	
*Acidic*					
3’-SL	0.18 ± 0.04 (24)	0.18 ± 0.05 (28)	0.17 ± 0.03 (18)	*p* = 0.736	
6’-SL	0.37 ± 0.15 (40)	0.39 ± 0.23 (59)	0.41 ± 0.15 (37)	*p* = 0.640	
LSTa	0.01 ± 0.01 (104)	0.01 ± 0.01 (90)	0.01 ± 0.00 (62)	*p* = 0.074	
LSTb	0.07 ± 0.04 (54)	0.10 ± 0.06 (58)	0.06 ± 0.03 (53)	*p* = 0.152	
LSTc	0.16 ± 0.09 (59)	0.13 ± 0.08 (64)	0.14 ± 0.06 (45)	*p* = 0.005	G1 = G3; G2 = G3; G2 < G1
*Total*					
Fucosylated ^c^	4.81 ± 1.62 (34)	2.07 ± 0.61 (29)	5.83 ± 1.97 (34)	*p* < 0.001	G1 = G3; G2 < G1; G2 < G3
Neutral core ^d^	0.50 ± 0.20 (41)	0.61 ± 0.26 (42)	0.46 ± 0.31 (67)	*p* = 0.243	
Acidic ^e^	0.79 ± 0.25 (31)	0.81 ± 0.35 (43)	0.79 ± 0.21 (27)	*p* = 0.245	
Total HMOs ^f^	6.10 ± 1.76 (29)	3.50 ± 0.84 (24)	7.08 ± 2.07 (29)	*p* < 0.001	G1 = G3; G2 < G1; G2 < G3

^a^ ANCOVA (Quade test) adjusted for postpartum days; ^b^ Tukey-Kramer test; ^c^ Sum of all individual fucosylated HMOs; ^d^ Sum of all individual neutral core HMOs; ^e^ Sum of all individual acidic HMOs; ^f^ Sum of all individual HMOs; G1: Group 1; G2: Group 2; G3: Group 3.

**Table 5:** Spearman rank correlation coefficient (r) between HMOs concentrations and quantitative clinical variables in Group 1 (Se+Le+; *n* = 59).

HMOs Concentrations	Maternal Variables							Infant Variables				
	Parity	Age	Postpartum Days	BMI	Weight	PG Weight	PG BMI	Gestational Age	Weight at Birth	Weight	Length	Weight Gain
*Fucosylated*												
2’-FL	−0.02	0.02	−0.07	0.18	0.20	0.14	0.18	−0.12	0.14	−0.05	0.11	−0.14
3’-FL	0.09	0.06	0.35	0.06	−0.09	0.04	−0.09	−0.07	−0.10	0.23	0.11	0.17
LNFP I	−0.07	−0.15	−0.47	0.05	0.11	0.01	0.05	0.09	0.08	−0.37	−0.14	−0.28
LNDFH I	−0.04	−0.21	−0.25	0.12	0.02	0.21	0.10	0.20	0.08	−0.29	0.04	−0.37
LNDFH II	0.07	0.04	0.27	−0.02	−0.13	0.01	−0.08	−0.14	−0.14	0.08	0.01	−0.02
DFLNHc	−0.08	0.01	−0.24	0.13	0.16	0.15	0.14	0.12	0.05	−0.06	0.07	0.04
DFpLNnH	0.05	−0.09	0.01	0.01	−0.03	0.12	0.08	−0.08	−0.20	−0.16	−0.22	−0.14
*Neutral core*												
LNH	−0.05	0.01	−0.42	0.02	0.17	−0.03	0.04	0.17	0.04	−0.20	−0.23	0.06
LNnH	−0.12	0.05	0.10	0.11	0.23	0.14	0.25	−0.04	0.06	0.09	0.02	0.07
LNT+LNnT	0.02	−0.15	−0.54	−0.13	−0.08	−0.13	−0.13	0.21	0.04	−0.39	−0.32	−0.13
*Acidic*												
3’-SL	−0.07	0.01	−0.25	−0.12	−0.10	−0.16	−0.18	−0.08	−0.17	−0.34	−0.32	−0.25
6’-SL	0.11	0.05	−0.71	−0.02	−0.07	−0.01	−0.09	0.22	0.07	−0.49	−0.25	−0.30
LSTa	−0.06	0.01	−0.62	−0.15	−0.12	−0.14	−0.17	0.25	0.01	−0.41	−0.29	−0.20
LSTb	0.05	−0.17	−0.26	−0.08	−0.07	−0.12	−0.11	0.01	0.03	−0.30	−0.22	−0.21
LSTc	0.05	−0.01	−0.76	−0.04	−0.07	0.02	−0.05	0.25	0.02	−0.54	−0.28	−0.36
*Total*												
												
Fucosylated	−0.10	−0.13	−0.24	0.14	0.13	0.13	0.13	0.05	0.11	−0.27	0.01	−0.32
Neutral core	−0.03	−0.07	−0.51	−0.07	0.04	−0.11	−0.07	0.25	0.09	−0.33	−0.32	−0.03
Acidic	0.05	0.02	−0.79	−0.07	−0.10	−0.05	−0.13	0.26	0.01	−0.61	−0.35	−0.42
Total HMOs	−0.06	−0.11	−0.43	0.15	0.13	0.14	0.11	0.10	0.13	−0.39	−0.10	−0.37

Significant correlations (*p* < 0.05) are highlighted in green (positive) and yellow (negative). PG: pre-gestational.

## Supplementary Materials

The following are available online at https://www.mdpi.com/2072-6643/11/6/1358/s1, Spreadsheet1: complete descriptive statistics of HMOs concentrations according to *SeLe* and *Se* phenotype.

## Appendix A

**Table A1:** Spearman rank correlation coefficient (r) between HMOs concentrations and quantitative clinical variables in Group 2 (Se−Le+; *n* = 9).

HMOs Concentrations	Maternal Variables							Infant Variables				
	Parity	Age	Postpartum Days	BMI	Weight	PG Weight	PPG BMI	Gestational Age	Weight at Birth	Weight	Length	Weight Gain
*Fucosylated*												
3’-FL	0.28	−0.06	0.35	−0.58	−0.52	−0.41	−0.14	−0.26	−0.14	0.46	0.49	0.77
LNDFH II	0.39	0.42	−0.08	−0.30	0.00	−0.32	0.11	−0.04	−0.29	−0.04	0.05	0.14
DFpLNnH	−0.06	−0.28	0.25	−0.02	−0.19	−0.28	−0.08	−0.18	−0.10	0.36	0.40	0.09
*Neutral core*												
LNH	−0.17	0.01	−0.50	0.08	−0.21	−0.08	−0.42	−0.54	−0.26	−0.56	−0.75	−0.06
LNnH	−0.17	−0.44	0.11	−0.32	−0.60	−0.32	−0.61	−0.49	−0.21	0.11	−0.02	0.26
LNT+LNnT	0.17	0.73	−0.35	0.00	0.19	−0.18	−0.16	0.22	−0.19	−0.43	−0.45	−0.49
*Acidic*												
3’-SL	0.62	0.38	−0.34	−0.08	0.05	−0.22	0.19	−0.22	−0.36	−0.46	−0.52	0.60
6’-SL	−0.06	0.89	−0.70	0.00	0.36	−0.05	0.12	−0.18	−0.50	−0.71	−0.72	−0.37
LSTa	−0.39	0.49	−0.69	0.40	0.45	0.41	0.12	−0.07	−0.19	−0.57	−0.58	−0.66
LSTb	0.28	0.37	−0.16	−0.37	−0.12	−0.72	−0.46	−0.11	−0.36	−0.46	−0.47	−0.31
LSTc	−0.39	0.45	−0.90	0.03	0.17	−0.29	−0.19	−0.72	−0.83	−0.75	−0.74	−0.49
*Total*												
Fucosylated	0.51	0.23	0.19	−0.57	−0.40	−0.47	−0.08	−0.10	−0.26	0.29	0.29	0.66
Neutral core	0.00	0.64	−0.46	0.02	0.05	−0.18	−0.40	0.02	−0.31	−0.38	−0.52	−0.41
												
Acidic	0.00	0.84	−0.66	0.03	0.32	−0.07	0.14	−0.19	−0.50	−0.61	−0.63	−0.20
Total HMOs	0.17	0.66	−0.38	−0.25	0.12	−0.25	0.11	−0.11	−0.55	−0.36	−0.31	0.09

Significant correlations (*p* < 0.05) are highlighted in green (positive correlations) and yellow (negative correlations). PG: pre-gestational.

**Table A2:** Spearman rank correlation coefficient (r) between HMOs concentrations and quantitative clinical variables in Group 3 (Se+Le−; *n* = 9).

HMOs Concentrations	Maternal Variables							Infant variables				
	Parity	Age	Postpartum Days	BMI	Weight	PG Weight	PG BMI	Gestational Age	Weight at Birth	Weight	Length	Weight Gain
*Fucosylated*												
2’-FL	−0.32	0.46	0.03	0.38	0.40	0.17	0.13	0.59	0.07	−0.04	0.04	0.07
3’-FL	−0.79	−0.23	−0.08	−0.28	−0.28	−0.50	−0.55	−0.05	−0.38	−0.57	−0.50	0.18
LNFP I	0.79	0.01	−0.12	0.28	0.38	0.48	0.68	−0.17	0.33	0.29	0.32	−0.61
DFpLNnH	0.00	−0.62	−0.03	0.23	0.13	0.13	−0.08	−0.37	0.00	0.04	0.09	−0.11
												
*Neutral core*												
LNH	0.79	−0.15	0.00	−0.20	−0.18	0.10	0.23	−0.48	0.21	0.21	−0.02	−0.36
LNnH	0.63	0.34	0.21	0.05	0.02	0.33	0.35	0.05	0.36	0.61	0.36	0.00
LNT+LNnT	0.79	−0.26	−0.11	−0.22	−0.13	0.03	0.18	−0.49	0.19	0.00	−0.05	−0.50
*Acidic*												
3’-SL	−0.32	−0.63	−0.38	−0.37	−0.28	−0.59	−0.50	−0.50	−0.43	−0.82	−0.59	0.07
6’-SL	−0.16	−0.16	−0.83	−0.02	0.28	0.06	0.48	−0.38	−0.45	−0.61	−0.81	−0.29
LSTa	0.79	−0.34	−0.09	−0.57	−0.53	−0.44	−0.15	−0.67	0.10	−0.04	−0.02	−0.46
LSTb	0.79	−0.55	0.09	0.32	0.25	0.30	0.23	−0.44	0.50	0.14	0.23	−0.82
LSTc	0.16	−0.79	−0.44	0.22	0.27	0.19	0.23	−0.69	−0.12	−0.21	−0.40	−0.75
												
												
*Total*												
Fucosylated	0.32	0.56	−0.15	0.37	0.53	0.40	0.55	0.39	0.40	0.04	−0.09	−0.32
Neutral core	0.80	−0.21	0.01	−0.19	−0.15	0.11	0.22	−0.44	0.26	0.25	0.10	−0.47
												
Acidic	−0.16	−0.48	−0.74	−0.18	0.09	−0.13	0.20	−0.55	−0.47	−0.67	−0.72	−0.41
Total HMOs	0.32	0.43	−0.22	0.43	0.63	0.57	0.75	0.28	0.33	−0.04	−0.13	−0.36

Significant correlations (*p* < 0.05) are highlighted in green (positive) and yellow (negative). PG: pre-gestational.

**Table A3:** Spearman rank correlation coefficient (r) between HMOs concentrations and quantitative clinical variables in Se+ women (*n* = 68).

HMOs Concentrations	Maternal Variables							Infant Variables				
	Parity	Age	Postpartum Days	BMI	Weight	PG Weight	PG BMI	Gestational Age	Weight at Birth	Weight	Length	Weight Gain
*Fucosylated*												
2’-FL	−0.04	0.00	−0.10	0.30	0.29	0.23	0.25	−0.04	0.11	−0.04	0.08	−0.15
3’-FL	0.09	0.10	0.31	−0.23	−0.14	−0.13	−0.20	−0.08	−0.09	0.16	0.07	0.17
LNFP I	−0.04	−0.18	−0.43	0.24	0.21	0.16	0.18	0.06	0.09	−0.30	−0.09	−0.28
LNDFH I	0.04	−0.04	−0.07	−0.08	−0.02	0.06	0.02	0.18	0.09	−0.19	0.11	−0.28
LNDFH II	0.10	0.09	0.29	−0.16	−0.09	−0.05	−0.10	−0.10	−0.10	0.09	0.05	0.00
DFLNHc	0.01	0.15	−0.08	0.03	−0.02	0.02	0.05	0.13	0.07	−0.03	0.13	0.04
DFpLNnH	0.07	−0.14	−0.01	−0.04	−0.02	0.07	0.05	−0.11	−0.16	−0.15	−0.17	−0.14
												
												
												
*Neutral core*												
LNH	0.05	0.04	−0.34	0.09	−0.03	−0.03	0.04	0.08	0.11	−0.15	−0.16	0.03
LNnH	−0.01	0.13	0.12	0.19	0.08	0.13	0.24	−0.02	0.13	0.13	0.09	0.06
LNT+LNnT	0.12	−0.14	−0.45	−0.10	−0.15	−0.12	−0.11	0.07	0.10	−0.35	−0.26	−0.14
*Acidic*												
3’-SL	−0.06	−0.03	−0.28	−0.14	−0.16	−0.22	−0.22	−0.12	−0.18	−0.37	−0.33	−0.24
6’-SL	0.07	0.04	−0.73	−0.02	−0.01	0.01	−0.03	0.14	0.00	−0.51	−0.31	−0.30
LSTa	0.01	0.03	−0.53	−0.18	−0.19	−0.18	−0.20	0.14	0.02	−0.39	−0.24	−0.21
LSTb	0.14	−0.22	−0.23	−0.04	−0.04	−0.07	−0.08	−0.06	0.11	−0.28	−0.16	−0.25
LSTc	0.06	−0.06	−0.72	−0.07	−0.06	0.00	−0.05	0.15	0.02	−0.53	−0.27	−0.39
												
												
*Total*												
Fucosylated	−0.06	−0.12	−0.28	0.24	0.25	0.22	0.21	0.08	0.12	−0.25	−0.01	−0.33
Neutral core	0.09	−0.07	−0.42	−0.01	−0.10	−0.10	−0.05	0.11	0.15	−0.29	−0.24	−0.07
												
Acidic	0.03	−0.03	−0.80	−0.08	−0.08	−0.07	−0.11	0.16	−0.03	−0.63	−0.39	−0.43
Total HMOs	−0.02	−0.11	−0.44	0.24	0.25	0.23	0.20	0.10	0.14	−0.37	−0.10	−0.39

Significant correlations (*p* < 0.05) are highlighted in green (positive) and yellow (negative). PG: pre-gestational.

**Table A4:** Spearman rank correlation coefficient (r) between HMOs concentrations and quantitative clinical variables in Se− women (*n* = 10).

HMOs Concentrations	Maternal Variables							Infant Variables				
	Parity	Age	Postpartum Days	BMI	Weight	PG Weight	PG BMI	Gestational Age	Weight at Birth	Weight	Length	Weight Gain
*Fucosylated*												
3’-FL	0.43	−0.24	0.18	−0.18	−0.36	−0.33	0.20	−0.37	0.07	0.31	0.32	0.29
LNDFH II	0.52	0.14	−0.22	0.22	−0.10	−0.29	0.38	−0.14	−0.02	−0.14	−0.08	−0.25
DFpLNnH	−0.09	−0.19	0.26	−0.42	−0.19	−0.37	−0.28	−0.10	−0.13	0.29	0.31	0.14
												
*Neutral core*												
LNH	−0.26	0.07	−0.35	−0.25	0.05	−0.03	−0.49	−0.43	−0.33	−0.43	−0.59	0.11
LNnH	−0.26	−0.28	0.18	−0.57	−0.35	−0.25	−0.62	−0.39	−0.30	0.12	0.01	0.36
LNT+LNnT	−0.09	0.78	−0.24	−0.10	−0.12	−0.18	−0.41	0.33	−0.28	−0.33	−0.35	−0.14
*Acidic*												
3’-SL	0.35	0.44	−0.28	−0.12	−0.22	−0.23	−0.12	−0.10	−0.35	−0.43	−0.46	0.57
6’-SL	0.09	0.70	−0.78	0.37	0.08	−0.11	0.14	−0.23	−0.43	−0.81	−0.81	−0.46
LSTa	−0.43	0.53	−0.51	0.15	0.24	0.37	−0.15	0.04	−0.28	−0.48	−0.48	−0.32
LSTb	0.09	0.54	−0.11	−0.23	−0.42	−0.65	−0.52	0.14	−0.35	−0.38	−0.38	−0.04
LSTc	−0.26	0.32	−0.91	0.15	0.07	−0.28	−0.18	−0.69	−0.75	−0.83	−0.83	−0.54
												
												
*Total*												
Fucosylated	0.61	−0.01	0.01	−0.08	−0.33	−0.39	0.24	−0.22	0.00	0.12	0.12	0.14
Neutral core	−0.26	0.68	−0.32	−0.20	−0.12	−0.16	−0.58	0.15	−0.45	−0.31	−0.40	−0.07
												
Acidic	−0.09	0.83	−0.63	0.23	0.07	−0.02	0.00	−0.01	−0.50	−0.55	−0.57	−0.11
Total HMOs	0.26	0.49	−0.50	0.17	−0.18	−0.33	0.16	−0.21	−0.43	−0.48	−0.44	−0.18

Significant correlations (*p* < 0.05) are highlighted in green (positive) and yellow (negative). PG: pre-gestational.

## References

[1] Dieterich C.M., Felice J.P., O’Sullivan E., Rasmussen K.M. (2013). Breastfeeding and health outcomes for the mother-infant dyad. Pediatr. Clin. North Am..

[2] Ballard O., Morrow A.L. (2013). Human milk composition: nutrients and bioactive factors. Pediatr. Clin. North Am..

[3] Newburg D.S., Neubauer S.H., Jensen R.G. (1995). Carbohydrates in milk: analysis, quantities and significance. Handbook of Milk Composition.

[4] Bode L. (2012). Human milk oligosaccharides: Every baby needs a sugar mama. Glycobiology.

[5] De Leoz M.L.A., Kalanetra K.M., Bokulich N.A., Strum J.S., Underwood M.A., German J.B., Mills D.A., Lebrilla C.B. (2015). Human Milk Glycomics and Gut Microbial Genomics in Infant Feces Show a Correlation between Human Milk Oligosaccharides and Gut Microbiota: A Proof-of-Concept Study. J. Proteome Res..

[6] Marcobal A., Barboza M., Froehlich J.W., Block D.E., German J.B., Lebrilla C.B., Mills D. (2010). a Consumption of human milk oligosaccharides by gut-related microbes. J. Agric. Food Chem..

[7] Asakuma S., Hatakeyama E., Urashima T., Yoshida E., Katayama T., Yamamoto K., Kumagai H., Ashida H., Hirose J., Kitaoka M. (2011). Physiology of consumption of human milk oligosaccharides by infant gut-associated bifidobacteria. J. Biol. Chem..

[8] Lin A.E., Autran C.A., Szyszka A., Escajadillo T., Huang M., Godula K., Prudden A.R., Boons G.-J., Lewis A.L., Doran K.S. (2017). Human milk oligosaccharides inhibit growth of group B Streptococcus. J. Biol. Chem..

[9] Newburg D.S., Ruiz-Palacios G.M., Morrow A.L. (2005). Human milk glycans protect infants against enteric pathogens. Annu. Rev. Nutr..

[10] Ruiz-Palacios G.M., Cervantes L.E., Ramos P., Chavez-Munguia B., Newburg D.S. (2003). Campylobacter jejuni Binds Intestinal H(O) Antigen (Fuc 1, 2Gal 1, 4GlcNAc), and Fucosyloligosaccharides of Human Milk Inhibit Its Binding and Infection. J. Biol. Chem..

[11] Morrow A.L., Ruiz-Palacios G.M., Altaye M., Jiang X., Lourdes Guerrero M., Meinzen-Derr J.K., Farkas T., Chaturvedi P., Pickering L.K., Newburg D.S. (2004). Human milk oligosaccharides are associated with protection against diarrhea in breast-fed infants. J. Pediatr..

[12] Donovan S.M., Comstock S.S. (2016). Human Milk Oligosaccharides Influence Neonatal Mucosal and Systemic Immunity. Ann. Nutr. Metab..

[13] Wang B. (2012). Molecular Mechanism Underlying Sialic Acid as an Essential Nutrient for Brain Development and Cognition. Adv. Nutr. An Int. Rev. J..

[14] Lis-Kuberka J., Orczyk-Pawiłowicz M. (2019). Sialylated Oligosaccharides and Glycoconjugates of Human Milk. The Impact on Infant and Newborn Protection, Development and Well-Being. Nutrients.

[15] Chen X. (2015). Human Milk Oligosaccharides (HMOS): Structure, Function, and Enzyme-Catalyzed Synthesis. Advances in Carbohydrate Chemistry and Biochemistry.

[16] Ninonuevo M.R., Park Y., Yin H., Zhang J., Ward R.E., Clowers B.H., German J.B., Freeman S.L., Killeen K., Grimm R. (2006). A strategy for annotating the human milk glycome. J. Agric. Food Chem..

[17] Zivkovic A.M., German J.B., Lebrilla C.B., Mills D.A. (2011). Human milk glycobiome and its impact on the infant gastrointestinal microbiota. Proc. Natl. Acad. Sci..

[18] Bode L., Jantscher-Krenn E. (2012). Structure-Function Relationships of Human Milk Oligosaccharides. Adv. Nutr. An Int. Rev. J..

[19] Kumazaki T., Yoshida A. (1984). Biochemical evidence that secretor gene, Se, is a structural gene encoding a specific fucosyltransferase. Proc. Natl. Acad. Sci..

[20] Johnson P.H., Watkins W.M. (1992). Purification of the Lewis blood-group gene associated a-3/4-fucosyltransferase from human milk: an enzyme transferring fucose primarily to Type 1 and lactose-based oligosaccharide chains. Glycoconj. J..

[21] Kobata A. (2010). Structures and application of oligosaccharides in human milk. Proc. Japan Acad. Ser. B.

[22] Thurl S., Henker J., Siegel M., Tovar K., Sawatzki G. (1997). Detection of four human milk groups with respect to Lewis blood group dependent oligosaccharides. Glycoconj. J..

[23] Thurl S., Munzert M., Henker J., Boehm G., Müller-Werner B., Jelinek J., Stahl B. (2010). Variation of human milk oligosaccharides in relation to milk groups and lactational periods. Br. J. Nutr..

[24] Musumeci M., Simpore J., D’Agata A., Sotgiu S., Musumeci S. (2006). Oligosaccharides in colostrum of Italian and Burkinabe women. J. Pediatr. Gastroenterol. Nutr..

[25] Gabrielli O., Zampini L., Galeazzi T., Padella L., Santoro L., Peila C., Giuliani F., Bertino E., Fabris C., Coppa G.V. (2011). Preterm milk oligosaccharides during the first month of lactation. Pediatrics.

[26] Kunz C., Meyer C., Collado M.C., Geiger L., García-Mantrana I., Bertua-Ríos B., Martínez-Costa C., Borsch C., Rudloff S. (2017). Influence of Gestational Age, Secretor, and Lewis Blood Group Status on the Oligosaccharide Content of Human Milk. J. Pediatr. Gastroenterol. Nutr..

[27] Elwakiel M., Hageman J.A., Wang W., Szeto I.M., van Goudoever J.B., Hettinga K.A., Schols H.A. (2018). Human Milk Oligosaccharides in Colostrum and Mature Milk of Chinese Mothers: Lewis Positive Secretor Subgroups. J. Agric. Food Chem..

[28] McGuire M.K., Meehan C.L., McGuire M.A., Williams J.E., Foster J., Sellen D.W., Kamau-Mbuthia E.W., Kamundia E.W., Mbugua S., Moore S.E. (2017). What’s normal? Oligosaccharide concentrations and profiles in milk produced by healthy women vary geographically. Am. J. Clin. Nutr..

[29] Azad M.B., Robertson B., Atakora F., Becker A.B., Subbarao P., Moraes T.J., Mandhane P.J., Turvey S.E., Lefebvre D.L., Sears M.R. (2018). Human Milk Oligosaccharide Concentrations Are Associated with Multiple Fixed and Modifiable Maternal Characteristics, Environmental Factors, and Feeding Practices. J. Nutr..

[30] Sprenger N., Odenwald H., Kukkonen A.K., Kuitunen M., Savilahti E., Kunz C. (2017). FUT2-dependent breast milk oligosaccharides and allergy at 2 and 5 years of age in infants with high hereditary allergy risk. Eur. J. Nutr..

[31] Bode L., Kuhn L., Kim H.-Y., Hsiao L., Nissan C., Sinkala M., Kankasa C., Mwiya M., Thea D.M., Aldrovandi G.M. (2012). Human milk oligosaccharide concentration and risk of postnatal transmission of HIV through breastfeeding. Am. J. Clin. Nutr..

[32] Kuhn L., Kim H.-Y., Hsiao L., Nissan C., Kankasa C., Mwiya M., Thea D.M., Aldrovandi G.M., Bode L. (2015). Oligosaccharide Composition of Breast Milk Influences Survival of Uninfected Children Born to HIV-Infected Mothers in Lusaka, Zambia. J. Nutr..

[33] Jantscher-Krenn E., Zherebtsov M., Nissan C., Goth K., Guner Y.S., Naidu N., Choudhury B., Grishin A.V., Ford H.R., Bode L. (2012). The human milk oligosaccharide disialyllacto-N-tetraose prevents necrotising enterocolitis in neonatal rats. Gut.

[34] Autran C.A., Kellman B.P., Kim J.H., Asztalos E., Blood A.B., Spence E.C.H., Patel A.L., Hou J., Lewis N.E., Bode L. (2018). Human milk oligosaccharide composition predicts risk of necrotising enterocolitis in preterm infants. Gut.

[35] Alderete T.L., Autran C., Brekke B.E., Knight R., Bode L., Goran M.I., Fields D.A. (2015). Associations between human milk oligosaccharides and infant body composition in the first 6 mo of life. Am. J. Clin. Nutr..

[36] Charbonneau M.R., O’Donnell D., Blanton L.V., Totten S.M., Davis J.C.C., Barratt M.J., Cheng J., Guruge J., Talcott M., Bain J.R. (2016). Sialylated Milk Oligosaccharides Promote Microbiota-Dependent Growth in Models of Infant Undernutrition. Cell.

[37] Associação Brasileira das Empresas de Pesquisa Critério de Classificação Econômica Brasil. http://www.abep.org/criterio-brasil.

[38] Vanna A.T., Yamada E., Arruda L.K., Naspitz C.K., Sole D. (2001). International Study of Asthma and Allergies in Childhood: Validation of the rhinitis symptom questionnaire and prevalence of rhinitis in schoolchildren in Sao Paulo, Brazil. Pediatr. Allergy Immunol..

[39] Solé D., Vanna A.T., Yamada E., Rizzo M.C., Naspitz C.K. (1998). International Study of Asthma and Allergies in Childhood (ISAAC) written questionnaire: Validation of the asthma component among Brazilian children. J. Investig. Allergol. Clin. Immunol..

[40] Yamada E., Vanna A.T., Naspitz C.K., Solé D. (2002). International Study of Asthma and Allergies in Childhood (ISAAC): Validation of the written questionnaire (eczema component) and prevalence of atopic eczema among Brazilian children. J. Investig. Allergol. Clin. Immunol..

[41] Tonon K.M., Miranda A., Abrão A.C.F.V., de Morais M.B., Morais T.B. (2019). Validation and application of a method for the simultaneous absolute quantification of 16 neutral and acidic human milk oligosaccharides by graphitized carbon liquid chromatography—Electrospray ionization—Mass spectrometry. Food Chem..

[42] Bai Y., Tao J., Zhou J., Fan Q., Liu M., Hu Y., Xu Y., Zhang L., Yuan J., Li W. (2018). Fucosylated Human Milk Oligosaccharides and N-Glycans in the Milk of Chinese Mothers Regulate the Gut Microbiome of Their Breast-Fed Infants during Different Lactation Stages. mSystems.

[43] Totten S.M., Zivkovic A.M., Wu S., Ngyuen U., Freeman S.L., Ruhaak L.R., Darboe M.K., German J.B., Prentice A.M., Lebrilla C.B. (2012). Comprehensive profiles of human milk oligosaccharides yield highly sensitive and specific markers for determining secretor status in lactating mothers. J. Proteome Res..

[44] Blank D., Dotz V., Geyer R., Kunz C. (2012). Human Milk Oligosaccharides and Lewis Blood Group: Individual High-Throughput Sample Profiling to Enhance Conclusions From Functional Studies. Adv. Nutr. An Int. Rev. J..

[45] Cangür Ş., Sungur M.A., Ankarali H. (2018). The Methods Used in Nonparametric Covariance Analysis. Duzce Tıp Fak Derg.

[46] Cangür Ş., Sungur M.A., Ankarali H. A web-based program for the Quade, Puri & Sen, and McSweeny & Porter Ranked ANCOVA Methods (Post Hoc Tukey-Kramer Test) for One-factor Covariance Model with Single-Covariate. http://www.masungur.com/nancova0.php.

[47] Thurl S., Munzert M., Boehm G., Matthews C., Stahl B. (2017). Systematic review of the concentrations of oligosaccharides in human milk. Nutr. Rev..

[48] Van Niekerk E., Autran C. (2014). Milk Oligosaccharides Differ between HIV-Infected and HIV-Uninfected Mothers and Are Related to Necrotizing Enterocolitis Incidence in Their Preterm Very-Low-Birth. J. Nutr..

[49] Seppo A.E., Autran C.A., Bode L., Järvinen K.M. (2017). Human milk oligosaccharides and development of cow’s milk allergy in infants. J. Allergy Clin. Immunol..

[50] King J.R., Varadé J., Hammarström L. (2018). Fucosyltransferase Gene Polymorphisms and Lewisb-Negative Status Are Frequent in Swedish Newborns, With Implications for Infectious Disease Susceptibility and Personalized Medicine. J. Pediatric Infect. Dis. Soc..

[51] Guo M., Luo G., Lu R., Shi W., Cheng H., Lu Y., Jin K., Yang C., Wang Z., Long J. (2017). Distribution of Lewis and Secretor polymorphisms and corresponding CA19-9 antigen expression in a Chinese population. FEBS Open Bio.

[52] Nordgren J., Sharma S., Bucardo F., Nasir W., Günaydin G., Ouermi D., Nitiema L.W., Becker-Dreps S., Simpore J., Hammarström L. (2014). Both lewis and secretor status mediate susceptibility to rotavirus infections in a rotavirus genotype-dependent manner. Clin. Infect. Dis..

[53] Corvelo T.C.O., Aguiar D.C.F., Sagica F.E.S. (2002). The expression of ABH and Lewis antigens in Brazilian semi-isolated Black communities. Genet. Mol. Biol..

[54] Bernardo C.R., Camargo A.V.S., Ronchi L.S., de Oliveira A.P., de Campos Júnior E., Borim A.A., Brandão de Mattos C.C., Bestetti R.B., de Mattos L.C. (2016). ABO, Secretor and Lewis histo-blood group systems influence the digestive form of Chagas disease. Infect. Genet. Evol..

[55] Coppa G.V., Gabrielli O., Zampini L., Galeazzi T., Ficcadenti A., Padella L., Santoro L., Soldi S., Carlucci A., Bertino E. (2011). Oligosaccharides in 4 different milk groups, Bifidobacteria, and Ruminococcus obeum. J. Pediatr. Gastroenterol. Nutr..

[56] Sprenger N., Lee L.Y., De Castro C.A., Steenhout P., Thakkar S.K. (2017). Longitudinal change of selected human milk oligosaccharides and association to infants’ growth, an observatory, single center, longitudinal cohort study. PLoS ONE.

[57] Erney R.M., Malone W.T., Skelding M.B., Marcon A.A., Kleman-Leyer K.M., O’Ryan M.L., Ruiz-Palacios G., Hilty M.D., Pickering L.K., Prieto P. (2000). A Variability of human milk neutral oligosaccharides in a diverse population. J. Pediatr. Gastroenterol. Nutr..

[58] Chaturvedi P., Warren C.D., Altaye M., Morrow A.L., Ruiz-Palacios G., Pickering L.K., Newburg D.S. (2001). Fucosylated human milk oligosaccharides vary between individuals and over the course of lactation. Glycobiology.

[59] Austin S., De Castro C., Bénet T., Hou Y., Sun H., Thakkar S., Vinyes-Pares G., Zhang Y., Wang P. (2016). Temporal Change of the Content of 10 Oligosaccharides in the Milk of Chinese Urban Mothers. Nutrients.

[60] Xu G., Davis J.C., Goonatilleke E., Smilowitz J.T., German J.B., Lebrilla C.B. (2017). Absolute Quantitation of Human Milk Oligosaccharides Reveals Phenotypic Variations during Lactation. J. Nutr..

[61] Doherty A.M., Lodge C.J., Dharmage S.C., Dai X., Bode L., Lowe A.J. (2018). Human Milk Oligosaccharides and Associations With Immune-Mediated Disease and Infection in Childhood: A Systematic Review. Front. Pediatr..

[62] Sjögren Y.M., Duchén K., Lindh F., Björkstén B., Sverremark-Ekström E. (2007). Neutral oligosaccharides in colostrum in relation to maternal allergy and allergy development in children up to 18 months of age. Pediatr. Allergy Immunol..

[63] Munblit D., Peroni D., Boix-Amorós A., Hsu P., Land B., Gay M., Kolotilina A., Skevaki C., Boyle R., Collado M. (2017). Human Milk and Allergic Diseases: An Unsolved Puzzle. Nutrients.

[64] Chen Y.-L., Chen J.-C., Lin T.-M., Huang T.-J., Wang S.-T., Lee M.-F., Wang J.-Y. (2005). ABO/secretor genetic complex is associated with the susceptibility of childhood asthma in Taiwan. Clin. Exp. Allergy.

[65] Innes A.L., McGrath K.W., Dougherty R.H., McCulloch C.E., Woodruff P.G., Seibold M.A., Okamoto K.S., Ingmundson K.J., Solon M.C., Carrington S.D. (2011). The H Antigen at Epithelial Surfaces Is Associated with Susceptibility to Asthma Exacerbation. Am. J. Respir. Crit. Care Med..

[66] Denborough M.A., Downing H.J. (1968). Secretor Status in Asthma and Hay Fever*. J. Med. Genet.

[67] Mandić Z., Pirički A.P., Kenjerić D., Haničar B., Tanasić I. (2011). Breast vs. bottle: Differences in the growth of Croatian infants. Matern. Child Nutr..

[68] Feldman-Winter L., Burnham L., Grossman X., Matlak S., Chen N., Merewood A. (2017). Weight gain in the first week of life predicts overweight at 2 years: A prospective cohort study. Matern. Child Nutr..

[69] Horta B., Victora C. (2013). Long-Term Health Effects of Breastfeeding: A Systematic Review (update).

[70] Vandenplas Y., Berger B., Carnielli V., Ksiazyk J., Lagström H., Sanchez Luna M., Migacheva N., Mosselmans J.-M., Picaud J.-C., Possner M. (2018). Human Milk Oligosaccharides: 2′-Fucosyllactose (2′-FL) and Lacto-N-Neotetraose (LNnT) in Infant Formula. Nutrients.

[71] Kunz C., Rudloff S., Isolauri E., Sherman P., Walker W. (2017). Compositional Analysis and Metabolism of Human Milk Oligosaccharides in Infants. Intestinal Microbiome: Functional Aspects in Health and Disease.

[72] Goehring K.C., Kennedy A.D., Prieto P.A., Buck R.H. (2014). Direct evidence for the presence of human milk oligosaccharides in the circulation of breastfed infants. PLoS ONE.

[73] Bhargava P., Li C., Stanya K.J., Jacobi D., Dai L., Liu S., Gangl M.R., Harn D.A., Lee C.-H. (2012). Immunomodulatory glycan LNFPIII alleviates hepatosteatosis and insulin resistance through direct and indirect control of metabolic pathways. Nat. Med..

[74] Wang M., Li M., Wu S., Lebrilla C.B., Chapkin R.S., Ivanov I., Donovan S.M. (2015). Fecal Microbiota Composition of Breast-Fed Infants Is Correlated With Human Milk Oligosaccharides Consumed. J. Pediatr. Gastroenterol. Nutr..

[75] Davis J.C.C., Lewis Z.T., Krishnan S., Bernstein R.M., Moore S.E., Prentice A.M., Mills D.A., Lebrilla C.B., Zivkovic A.M. (2017). Growth and Morbidity of Gambian Infants are Influenced by Maternal Milk Oligosaccharides and Infant Gut Microbiota. Sci. Rep..

